# A Case of Fractured Ribs Which Terminated Fatally; with the Appearances on Dissection, and Remarks

**Published:** 1790

**Authors:** George Wilkinson

**Affiliations:** Surgeon at Sunderland, Member of the Royal College of Surgeons, and Honorary Member of the Chirurgo-physical Society of Edinburgh


III. *A Case of fractured Ribs which terminated fatally; with the Appearances on Dissection, and Remarks. Communicated in a Letter to Dr.* Simmons, *F. R. S. by Mr.* George Wilkinson, *Surgeon at Sunderland, Member of the Royal College of Surgeons, and Honorary Member of the Chirurgo-physical Society of Edinburgh.*

ON Tuesday, the 10th of February, 1789, I was desired to visit Mr. J. H., an officer of Excise, aged fifty-six years, and of a robust constitution, who had the preceding day received a fall on his left side against the edge of a ladder.

I examined his side with attention, but could not then discover any appearance of fracture, though it was on the sixth and seventh ribs that he complained much of being sore and painful.

He was bled freely, his ribs were encompassed with a flannel roller, a spermaceti emulsion, with sal. vol. c. c. and tinct. opii camph.

was directed at proper intervals; and a camphorated volatile liniment was rubbed upon the part affected.

By this mode of treatment, which was continued for the space of eight or nine days, he grew infinitely better, having little or no cough, nor any appearance of inflammation. His pulse, at the same time, was pretty regular, and his belly naturally open.

On Friday, the 20th, being the twelfth day from his receiving the fall, he found himself so well recovered, that, notwithstanding the weather was cold and windy, he ventured (though without my permission) into the open air, remained out about two hours, and walked upwards of two miles.

Sunday, the 22d, I was called to see him, and found he had suffered from his temerity, being now seized with a dry cough and thirst: his pulse was quick, though somewhat soft and feeble; and his breathing was rather difficult, with a sense of weight about the thorax, though he found himself free from pain, particularly in the part injured.

He was ordered the lac. ammon., with oxymel of squills, and tinct. opii camphorat.

The fucceeding day he was much worfe, his refpiration was laborious, and his cough violent, with little expectoration, except now and then a very fmall quantity of vifcid mucus, which, on being fpit into a glafs of water, funk to the bottom.

It was not till this day that I difcovered he had neglected to wear a flannel fhirt I had recommended in the beginning of his complaint. It was now, however, (though too late) adopted: a blifter was at the fame time applied between the fhoulders, and the fpermaceti draughts, with the addition of vin. antim., were adminiftered at proper intervals.

On Tuefday, the 24th, the difficulty of breathing was increafed, and accompanied with ftertor; his countenance was palid, his eyes appeared funk, and the blifter had affected him with a troublefome ftrangury, his urine being much tinged with blood.

Next day, being the 25th, about ten o'clock in the morning, fixteen days from his receiving the accident, and the fifth or fixth from his venturing out into the open air, he took a breakfaft of barley gruel, put on his cloaths, rofe out of bed, and a very fhort time after fuddenly expired.

During

During the whole courſe of his diſorder his belly was regular, his appetite tolerably good, and he could lay equally well on either ſide.

The body was opened next day in the preſence, and with the aſſiſtance, of Mr. Akenhead, an ingenious ſurgeon of this place, and another gentleman.

On carefully examining the cavity of the thorax the lungs appeared of a dark blue, or rather black colour, evidently approaching to ſphacelus. Their ſubſtance was extremely ſoft and tender, and, on being cut into, we found the blood of a diſſolved texture, diffuſed through the whole of their cellular ſpaces, and in many parts their external membrane appeared detached from its adheſion, and raiſed in the manner of veſicles. The bronchial ſyſtem, however, contained very little mucus; the pericardium but little water; the heart and its veſſels, as well as the pulmonary artery of the lungs, ſeemed perfectly ſound; the right auricle was much diſtended with black blood; and what greatly ſurpriſed us was, that not the leaſt exſudation or adheſion had taken place in the cavity of the thorax, and that no appearance of inflammation could be ſeen on any part of the pleura.

The

The parts fractured were thofe of the fixth and feventh ribs of the left fide, about a hand's breadth or more from their articulation with the fpine, and broke off fo very fhort and tranfverfely as fcarcely to be feparable even with a degree of force applied.

Not the leaft appearance of difcoloration was to be feen internally on the parts fractured, and the whole of the lungs evidently appeared to us to have been free from any previous difeafe.

In the cavity of the abdomen the whole of the vifcera appeared perfectly found; the kidnies, bladder, &c., being quite free from any effects which had produced the bloody urine and ftrangury.

From a review of the cafe juft recited, together with the appearances on diffection, does it not feem probable that the patient's death was occafioned by pneumonic inflammation terminating in gangrene? The effufion of blood into the cellular fpaces of the lungs, together with the difeafe being deeply feated in their fubftance, the total abfence of morbid appearances (fuch as adhefion, exfudation of coagulable lymph, &c.) in other parts, and the found ftate of of the *pleura coſtalis*, are all of them circumſtances that tend to corroborate the idea of its being an idiopathic affection of that viſcus.

Notwithſtanding it has been queſtioned by the great and juſtly celebrated Dr. Cullen, whether this diſeaſe ever originates in the parenchyma or cellular texture of the lungs, on a ſuppoſition that it conſtantly begins in their external membrane, or ſome part of the pleura*; yet it would appear, that, in the caſe before us, ſuch inflammation did take its riſe in the parenchyma. The abſence of pain from the commencement of its attack, which ſeems to be a leading ſymptom when the pleura or membranes are the ſeat of the diſeaſe, was in this inſtance marked only by a ſenſe of weight or fulneſs, it being a well-known fact that the ſubſtance of the lungs, when in a ſtate of inflammation, is by no means acutely ſenſible; though indeed the injury which the patient received by the fall, with the pain and ſoreneſs that at firſt attended it, would have naturally led us to expect to find ſome marks of diſeaſe in the pleura on diſſection.

* Firſt Lines, Vol. I. art. 342.

Indeed it muft be owned, that though this fpecies of inflammation is lefs frequent than that which takes place in the pleura or membrane invefting the lungs, yet it probably happens oftener than we are aware of; and I have been led to this opinion from what has come under my obfervation in thofe patients who have been fuddenly carried off by the malignant meafles, the appearances of whofe diffections, as defcribed by the late ingenious Sir William Watfon *, feem to corroborate this fact.

As a farther proof of the probability of the truth of this doctrine, I need only mention a cafe which happened to me fome years ago, in which the patient died fuddenly of a pulmonary affection. Upon opening the cavity of the thorax, neither the pleura coftalis, nor that covering the lungs, were in any wife affected, but nearly the whole of both lungs was converted into pus, which, in this cafe, produced a fatal fuffocation, though the patient fpat pus before this happened.

By many it will, I have no doubt, be fuppofed that this difeafe was occafioned by the fracture of the ribs, or produced by a metaf-

* Medical Obf. and Inq. Vol. IV. page 432.

tafis. But, I muft confefs, that, to me, the neglect of wearing the flannel fhirt, together with the patient's imprudent expofure of himfelf to the cold air, and the fatigue he experienced in walking, are fufficient to account for the commencement of the difeafe.

I am well aware that pulmonary inflammation often goes on very obfcurely, and is with difficulty afcertained; but if the fracture of the ribs had been the caufe of the fatal termination of this cafe, there muft furely have been difcovered fome marks of difeafe on the pleura, fome appearance of inflammation or exfudation on its furface. Nothing of this fort, however, could be difcovered, though the body was examined in a good light, and with great care and attention. But whatever difference of opinion may take place with refpect to the theory of this cafe, the circumftances that attended it will, I flatter myfelf, be confidered, Sir, by you as curious. I have related them faithfully, and fubmit them, with great deference, to your judgment.

*Sunderland,*

March 2, 1790.

